# Phage derived lytic peptides, a secret weapon against *Acinetobacter baumannii*—An *in silico* approach

**DOI:** 10.3389/fmed.2022.1047752

**Published:** 2022-11-04

**Authors:** Abhishek Nandy, Ruchi Yadav, Aditi Singh

**Affiliations:** Amity Institute of Biotechnology, Amity University Uttar Pradesh, Lucknow, India

**Keywords:** endolysin, antimicrobial peptides, biofilm, anti-fungal, *Acinetobacter baumannii*, AMP

## Abstract

*Acinetobacter baumannii* is a bacterial pathogen that is commonly associated with hospital-acquired illnesses. Antimicrobial drug resistance in *A. baumannii* includes several penicillin classes, first and second-generation cephalosporins, cephamycins, most aminoglycosides, chloramphenicol, and tetracyclines. The recent rise in multidrug-resistant *A. baumannii* strains has resulted in an increase in pneumoniae associated with ventilators, urinary tract infections associated with the catheter, and bloodstream infections, all of which have increased complications in treatment, cost of treatment, and death. Small compounds known as antimicrobial peptides (AMPs) are known to have damaging effects on pathogenic bacteria. To determine their antimicrobial activity, AMPs are created from proteins acquired from various sources and evaluated *in vitro*. In the last phase of lytic cycle, bacteriophages release hydrolytic enzymes called endolysins that cleave the host’s cell wall. Due to their superior potency and specificity compared to antibiotics, lysins are used as antibacterial agents. In the present study, different types of endolysin from phages of *A. baumannii* were selected based on an extensive literature survey. From the PhaLP database, the sequences of the selected lysins were retrieved in FASTA format and antimicrobial peptides were found among them. With the help of available bioinformatic tools, the anti-biofilm property, anti-fungal property, cell-penetrating property, and cellular toxicity of the antimicrobial peptides were determined. Out of the fourteen antimicrobial peptides found from the eight selected endolysins of *A. baumannii* specific phage, eight of them has anti-biofilm property, nine of them has anti-fungal property, five of them has cell-penetrating property and all of them are non-toxic.

## Introduction

*Acinetobacter baumannii* is gram-negative, non-motile and aerobic coccobacillus. The six “ESKAPE” pathogens with multidrug resistance and pathogenicity include *A. baumannii*. This group oversees most nosocomial illnesses and can avoid the biocidal effects of antibiotics (1, 2). With a 2–10% death rate documented among patients with chronic UTIs, bacteremia, pneumonia and critically sick patients in ICU, *A. baumannii* has quickly established itself as the most successful nosocomial pathogen (3). A major area of risk is the emergence and spreading of Acinetobacter species, which are resistant to most antimicrobial drugs now on the market (4). The World Health Organization has issued a red alert regarding the “critical” category-designated carbapenem-resistant *A. baumannii* (5). As a result, infections caused by the drug-resistant pathogen *A. baumannii* become challenging to treat. It can infect injuries in different areas of the body as well as the blood, urinary tract, and lungs (pneumonia) (6). Treatment complications, costs, and mortality in pneumoniae associated with ventilators, sepsis, and infections of the urinary tract associated with catheters, have increased because of an upsurge in multidrug-resistant *A. baumannii* strains. One of the strategies to counter this growing antimicrobial resistance in recent times is to fight it with phage therapy, which is using bacteriophage to fight antibiotic-resistant bacteria.

Bacteriophages, or phages as they are commonly known, are viruses that only infect and replicate in bacterial cells (7). They are regarded as the most prevalent biological agent on earth and are widely distributed throughout the environment. Their size, appearance, and genetic organization are incredibly different (8). They are stable in a variety of conditions and highly specialized to the bacterial host. Phages have a remarkably wide range of structural, physicochemical, and biological characteristics. Bacteriophages with tails make up more than 95% of all known phages; the other 2% are polyhedral, filamentous, and pleomorphic with genomes that can be single/double-stranded RNA, or single/double-stranded DNA (9, 10). Bacteriophages can also be divided into groups based on how they manage to get away from their hosts, such as lytic phages and filamentous phages (11). They are very selective and will recognize bacteria from a single species or strain. Because it binds to a specific receptor on the surface of the host, bacterial phage identification is specific. The phage binds to the bacterial cell after identifying this receptor (12).

Since almost a century ago, phage therapy has been used to treat bacterial infections by using bacterial viruses (phages). Before the development of antibiotics, many bacterial infections in various Eastern European nations were treated with bacteriophages (13). The first documented clinical application of phages was made in 1919 at the Hôpital des Enfants-Malades in Paris, where phages were successfully employed to treat four pediatric instances of bacterial diarrhea. It was Felix d’Herelle who had the notion to use phages therapeutically (14), however, due to complications in clinical trials in the early days and the discovery of antibiotics, it was not accepted by Western Countries (15). In recent times, the focus on phage therapy has again increased in view of growing antibiotic resistance and the approach has been investigated against a variety of pathogens using animal models.

Every bacteriophage consists of a nucleic acid molecule encased in a protein framework. A bacteriophage infects a host cell by attaching to a receptor on the bacterial strain that is vulnerable to it. When a bacterium becomes infected, a bacteriophage seizes control of its host’s cellular machinery and drives the cell to create viral components rather than bacterial ones. Eventually, a process known as lysis causes additional bacteriophages to form and erupt from the bacterium (16). These phages frequently employ an enzyme called Phage lytic proteins to lyse the host. Unlike antibiotics that may kill any type of bacteria, these phage lysins, a group of unrelated antimicrobial peptides (AMPs) cause hydrolysis of bacterial cell wall thus acting as a guided missile to kill it (17). Such AMPs, which are a component of innate immunity in living things, have activity against a variety of bacteria and are found in abundance in nature to play a crucial role in the innate immune systems of various animals. With regard to bacteria, fungi and parasites, AMPs have a variety of inhibitory actions. Based on their structural characteristics, antimicrobial peptides can be classified into four groups: linear -helical peptides, -sheet peptides, linear extension structures, and both -helix and -sheet peptides. In addition, more complicated cyclic peptides and AMPs (such as lasso peptides and thioether bridged structures) are also observed (18, 19). The AMPs have a promising application possibility in medicine, food, animal husbandry, agriculture, and aquaculture, and exploratory studies on them were prompted by the advent of antibiotic-resistant microbes and the growing worries about the use of antibiotics (20). Therefore, one of the most cutting-edge alternative antibacterial now being studied in clinical trials are such phage lytic AMPs. These bacterial peptidoglycan-degrading enzymes, which are produced by bacteriophages, cause instantaneous cell death. However, there aren’t many reports on how bacteriophages, phage endolysins, and antimicrobial peptides interact with harmful bacteria directly. A synergistic effect was seen in each of them, with phage-encoded antimicrobial peptides serving as a particular class of AMPs (21). The ability of phage lytic proteins to be viewed as a novel class of antimicrobials, dubbed enzybiotics, with the capacity to target any bacterial infection, is one of its most significant characteristics. To fully use the rich natural variety, additional phage lytic enzyme discoveries are still necessary (22, 23).

The lysins of bacteriophage not only possess the property to disrupt cell wall but also have antibiofilm properties. The biofilm-associated diseases that *A. baumannii* frequently causes, like catheter-related infections and pneumonia linked with ventilators, are both resistant to antibiotic treatment (24). High doses of antibiotics are often needed to break through dense biofilms to see any bacterial growth inhibition, but total eradication is uncommon. However, phage lytic proteins have the ability not only to inhibit the biofilm formation, but also its growth in addition to eradicating biofilms (25–27). According to certain theories, bacteriophage could significantly affect the growth of fungi (28). *Aspergillus fumigatus* and *Candida albicans* biofilms have recently been found to be inhibited by the bacteriophages of *Pseudomonas aeruginosa* (29).

There is a lack of data that focuses on the safety profile of endolysins, despite the fact that much research is being done on how effective they are against bacterial targets. Similarly, there is a lack of information on the pharmacokinetics and pharmacodynamics of endolysins as possible medications. Pharmacodynamics examines how a medicine affects the body, while pharmacokinetics focuses on what the body does to a specific substance. The approval of a medication for therapeutic use depends on each of these considerations (23, 30). Therefore, some properties of the phage lytic proteins like cellular toxicity and cell-penetrating property should be tested before administering these enzymes to humans for the treatment of bacterial infections.

## Methodology

### Selection of phage peptide sequence

Different bacteriophages specific to *A. baumannii* were selected and their peptide sequences were downloaded from PhalP Database in FASTA format. The selected lytic peptides are mentioned in Table 1, along with their mode of action (31).

**TABLE 1:** Selected classes of phage lytic proteins against *Acinetobacter baumannii*.

Sr. no	Protein name	Mode of action	References
1.	*N*-acetylmuramidase	Hydrolysis of terminal, non-reducing *N*-acetylmuramic residues.	(23, 28)
2.	Lysozyme	Disrupts the peptidoglycan cell wall’s structural stability by hydrolyzing the 1,4 glycosidic bonds between NAG and NAM monomers of the peptidoglycan polymers.	(29, 30)
3.	Endolysin	Catalyze the hydrolysis of the glycosidic linkage.	(19, 23)
4.	Glycosylhydrolase	Catalyzes the hydrolysis of glycosidic bonds of *O*- or *S*-glycosides.	(30)
5.	Carboxypeptidase	Cleaves mainly free carboxyl groups next to the peptide or ester link on *L*-amino acids.	(19, 49)
6.	Endopeptidase	Break the bonds between the terminal peptide’s two amino acids. The Interpeptide bridge or terminal peptide-interpeptide bridge can both experience bond cleavage.	(30, 50)
7.	Peptidoglycan binding protein	Innate immune system pattern-recognition receptors that bind and, in some situations, hydrolyze bacterial peptidoglycan	(49, 51)

### Antimicrobial peptide identification

A web-based tool called Antimicrobial Peptide Analysis (AMPA) is used to locate and evaluate a protein’s antimicrobial domains. We have used the same resource to predict and create peptide-based medications having anti-microbial effects (21). For prediction, one or more peptide sequences in FASTA format are provided. In order to numerically represent the amino acids (AA) of a peptide with respect to various functional regions, the server has first calculated “features” (a combination of physicochemical properties and designed sequence motifs): global (average of all AA), N-terminal (average of the first 10 AA), amphipathic-segment (average of the most amphipathic window, 10-18 AA in length), and C-terminal (average of the last 10 AA). Each of these locations has been demonstrated to play a significant role for AMP activity, and the dissertation provides information on the particular qualities taken into account.

### Anti-biofilm property

To identify the anti-biofilm property of selected proteins, we have used the dPABB tool. The dPABB (design Peptides Against Bacterial Biofilms) algorithm identifies anti-biofilm peptides, based on the amino acid composition, chosen residue, and position of the residues. The predicted antimicrobial peptides were pasted in the webpage and on the basis of their entire amino acid composition, specific residue characteristics, and the positional preference of the residues, the six-support vector machine (SVM) and Weka models deployed on dPABBs were observed to find anti-biofilm peptides. The anti-biofilm property is then determined using the scores generated for each of the peptide compounds (18).

### Antifungal property

To predict the antifungal properties of antimicrobial peptides, we have used the Antifp tool. It is a freely available software used for *in silico* studies, through which users can forecast one or more sequences for the module’s antifungal properties. Using the program, we searched protein sequences for peptides and their mutant analogues, then screen the results for antifungal properties (32). Support vector machine (SVM)-based models is used for predicting the antifungal property of the antimicrobial peptides.

### Cell penetrating property

High throughput techniques have been used to identify novel peptide compounds that can enter cells. Therefore, *in silico* screening approaches combined with experimental validation are considered more practical and economical. The outcomes could be reliably and easily repeated in “*in vitro*” settings. One such standalone program created to forecast and create peptide compounds that penetrate cells is CellPPD (33), which has been used in our study to assess the cell-penetrating properties of our selected peptides. The predicted antimicrobial peptides were pasted in the webpage and Support vector machine (SVM)-based models is used for predicting and designing highly effective cell penetrating peptides.

### Toxicity prediction

Designing antimicrobial peptides requires careful consideration of peptide toxicity. In the current investigation, we have made use of the ToxinPred tool. The program recognizes specific residues of certain amino acids, including Cys, His, Asn, and Pro, and their placements at different places that render them poisonous. ToxinPred can be used to forecast the toxicity or non-toxicity of a proposed peptide, the effects of mutations on toxicity, and the location of toxic protein regions (34, 35). Multiple Em for Motif Elicitation (MEME, version 4.9.0) program for the identification of prominent motifs in the toxic peptides.

## Results and discussion

From the different types of endolysins of *A. baumannii* specific phage, the current analysis predicts antimicrobial peptides. These peptides were found to have anti-biofilm and anti-fungal effects too. Most of the AMPs were discovered to be non-toxic and non-cell penetrating.

*In silico* prediction tools identified AMPs in all eight selected endolysins. These endolysins are present in those phages which are specific for *A. baumannii.* The phage lytic protein, named Virion Associated Lysins (VAL) was found to have a maximum of three different AMP molecules, as identified and predicted in our study. Similarly, Endolysin, Chitinase, Carboxypeptidase, and Murein transglycosylase were identified for having two different AMPs each, however in PG_binding_3 domain, 1,4-beta-*N*-acetylmuramidase and Lysozyme we could find only one potential AMP molecule. One peptide among each type of following phage lytic protein was predicted to exhibit anti-biofilm property. All the peptides of VAL, Endolysin, Chitinase and one peptide of Carboxypeptidase and Murein transglycosylase was predicted to possess anti-fungal property. One peptide of VAL, Endolysin, Chitinase, Carboxypeptidase, and Murein transglycosylase was predicted to have cell-penetrating property.

The various classes of phage lytic proteins against *A. baumannii* included in the study are presented in Table 2. Almost all the tested peptides were predicted to be non-toxic in nature. So, the peptides will have high therapeutic value and will not interfere with the human immune response. Protein name with its Uniprot accession number, Name of the Phage, Name of the host and the predicted Antimicrobial Peptide is also mentioned in Table 2.

**TABLE 2:** Predicted antimicrobial peptides from the protein sequences of *Acinetobacter baumannii* specific phage.

S. no.	Protein name	Uniprot accession	Phage name	Host name	Antimicrobial peptide	Type
	Virion associated lysins (VAL)	A0A0P0IE11	Acinetobacter phage Ab105-1phi	*Acinetobacter baumannii*	STIKKSVSKSYG	I
					MTKIGKRFRTKN	II
					QYQKGRFKKHYNSAN	III
	PG_binding_3 domain-containing protein	A0A7G3T113	Acinetobacter phage Ab11510-phi	*Acinetobacter baumannii*	LVRVLNIMQGQR	
	Putative endolysin	L7TMD3	Acinetobacter phage AB3	*Acinetobacter baumannii*	SKKYYPYIGYGY	I
					GFRRKRPVSKYNKQQYIA	II
4.	Putative chitinase	L7TK19	Acinetobacter phage AB3	*Acinetobacter baumannii*	SKKYYPYIGYGY	I
					GFRRKRPVSKYNKQQYIA	II
5.	Carboxypeptidase	A0A3Q9R756	Acinetobacter phage AbKT21phiIII	*Acinetobacter baumannii*	SKKYYPYIGYGY	I
					FRRKRPVSKYNKQQYIA	II
6.	1,4-beta-*N*-acetylmuramidase	A0A346FIJ7	Acinetobacter phage ABPH49	*Acinetobacter baumannii*	KGRKSKVINSKGL	
7.	Putative lytic murein transglycosylase	A0A6B9STC3	Acinetobacter phage vB_AbaM_Lazarus	*Acinetobacter baumannii*	NMQKAYKYGRSQPL	I
					GWKHQRGALYSRNVLKKANY	II
8.	Lysozyme	A0A0B5L1J1	Acinetobacter phage RL-2015	*Acinetobacter baumannii*	LKYKYVAKRDCS	

All the antimicrobial properties like anti-biofilm, anti-fungal, cell-penetrating properties and cellular toxicity of the predicted peptides are represented in Table 3. All peptides showed variable potential for these parameters; however only three of the tested peptides, namely Virion associated lysin II, Putative endolysin II, and Putative chitinase II were positive for all parameters. These three peptides were found to be possessing anti-biofilm, anti-fungal, and cell-penetrating capacity in our *in silico* study and can be explored further.

**TABLE 3:** Predicted antimicrobial peptides and their anti-biofilm and anti-fungal properties.

Protein name	Type	Antimicrobial peptide	Anti-biofilm property	SVM score	Anti-fungal property	Score	Cell penetrating property	Toxicity
Virion associated lysins (VAL)	I	STIKKSVSKSYG	Inactive	–0.79	Antifungal	0.50740539	Non-CPP	Non-toxic
	II	MTKIGKRFRTKN	Active	0.36	Antifungal	1.1083252	CPP	Non-toxic
	III	QYQKGRFKKHYNSAN	Inactive	–0.10	Antifungal	0.1585372	Non-CPP	Non-toxic
PG_binding_3 domain-containing protein		LVRVLNIMQGQR	Active	0.90	Non-antifungal	0.44300579	Non-CPP	Non-toxic
Putative endolysin	I	SKKYYPYIGYGY	Inactive	–0.75	Antifungal	0.23495578	Non-CPP	Non-toxic
	II	GFRRKRPVSKYNKQQYIA	Active	0.57	Antifungal	0.136447	CPP	Non-toxic
Putative chitinase	I	SKKYYPYIGYGY	Inactive	–0.75	Antifungal	0.23495578	Non-CPP	Non-toxic
	II	GFRRKRPVSKYNKQQYIA	Active	0.57	Antifungal	0.136447	CPP	Non-toxic
Carboxypeptidase	I	SKKYYPYIGYGY	Inactive	–0.75	Antifungal	0.23495578	Non-CPP	Non-toxic
	II	FRRKRPVSKYNKQQYIA	Active	0.47	Non-antifungal	0.070030118	CPP	Non-toxic
1,4-beta-*N*-acetylmuramidase		KGRKSKVINSKGL	Active	0.96	Non-antifungal	0.58543309	Non-CPP	Non-toxic
Putative lytic murein transglycosylase	I	NMQKAYKYGRSQPL	Inactive	–0.54	Antifungal	0.1298745	Non-CPP	Non-toxic
	II	GWKHQRGALYSRNVLKKANY	Active	1.25	Non-antifungal	0.21096256	CPP	Non-toxic
Lysozyme		LKYKYVAKRDCS	Active	0.11	Non-antifungal	0.059544883	Non-CPP	Non-toxic

Physio-chemical properties like Hydrophobicity, Hydrophilicity and Molecular weight of the predicted antimicrobial peptides are essential for drug design as it is a key factor for the formulation of the drug and its interaction with the human body. The physiochemical properties cause the receptor—which may be a biological molecule or system—to respond pharmacologically. All the physicochemical properties of the predicted antimicrobial peptides are represented in Table 4, with PG_binding_3 domain-containing protein and 1,4-beta-*N*-acetylmuramidase having the lowest molecular weight.

**TABLE 4:** Physiochemical properties of the antimicrobial peptides.

Protein name	Type	Antimicrobial peptide	Hydrophobicity	Hydropathicity	Hydrophilicity	Molecular weight
Virion associated lysins (VAL)	I	STIKKSVSKSYG	–0.26	–0.72	0.35	1284.64
	II	MTKIGKRFRTKN	–0.51	–1.40	0.73	1479.98
	III	QYQKGRFKKHYNSAN	–0.49	–2.17	0.33	1869.29
PG_binding_3 domain-containing protein		LVRVLNIMQGQR	–0.19	0.21	–0.26	1426.94
Putative endolysin	I	SKKYYPYIGYGY	–0.11	–1.08	–0.58	1501.87
	II	GFRRKRPVSKYNKQQYIA	–0.48	–1.54	0.44	2239.87
Putative chitinase	I	SKKYYPYIGYGY	–0.11	–1.08	–0.58	1501.87
	II	GFRRKRPVSKYNKQQYIA	–0.48	–1.54	0.44	2239.87
Carboxypeptidase	I	SKKYYPYIGYGY	–0.11	–1.08	–0.58	1501.87
	II	FRRKRPVSKYNKQQYIA	–0.52	–1.61	0.47	2182.80
1,4-beta-*N*-acetylmuramidase		KGRKSKVINSKGL	–0.40	–1.04	0.82	1414.91
Putative lytic murein transglycosylase	I	NMQKAYKYGRSQPL	–0.36	–1.48	0.12	1684.15
	II	GWKHQRGALYSRNVLKKANY	–0.33	–1.20	0.07	2390.05
Lysozyme		LKYKYVAKRDCS	–0.39	–0.90	0.49	1473.91

Antibiotic effectiveness is in jeopardy due to the worldwide rapid growth of resistant microorganisms. Antibiotic development and use have, predictably, contributed to the current issue of escalating antibiotic resistance and waning new treatments (36, 37). The removal of these multidrug-resistant organisms from the infection site is still a challenging process that necessitates the use of antibiotics. The rise of drug-resistant versions of these infections has increased interest in finding new treatment targets for them (38). Lysins have been utilized successfully to manage pathogenic, antibiotic-resistant bacteria present on mucosal membranes and diseased tissues in a range of animal models. Experimental models of sepsis, pneumonia, meningitis, endocarditis, and mucosal colonization show that lysins may remove bacteria from mucosal surfaces and biofilms both systemically and topically. Their ability to target the disease specifically without affecting the normal flora, low risk of bacterial resistance, with their hitherto unattainable ability to kill colonizing infections on mucosal surfaces make them preferable to antibiotics (39).

Numerous natural and artificial AMPs, including nisin, cecropins, and defensins, have demonstrated effective suppression of both Gram-positive and Gram-negative bacteria (40). A subgroup of AMPs known as antifungal peptides (AFPs) treats fungal infections with increased treatment resistance. Numerous AFPs have demonstrated excellent anti-fungal activity against common pathogenic fungi, including yeast, filamentous fungus (such *Aspergillus flavus*), mould, and *Aspergillus* and *Candida albicans* in clinical practise (41). Antimicrobial peptides are promising to be anti–biofilm agents. Anti-biofilm peptides can treat chronic multi-resistant bacterial infections by targeting biofilms using a variety of mechanisms, such as signal degradation within biofilms, permeabilization within cytoplasmic membrane/EPS, controlling EPS synthesis, etc. (42, 43). However, there can be shortcomings too with AMPs and the manufactures are constrained by some factors, like AMPs can harm eukaryotic cell membranes and have hemolytic side effects, their high manufacturing costs and technical difficulties in production, their limited consistency at certain pH. reduced activity in the presence of iron, easy digestibility by proteases (44). These aspects need attention before their application as therapeutic molecule.

In the past two decades, lysins’ capacity to stop pathogenic colonization of the mucosa has been demonstrated by several promising pre-clinical trials, the first of which was conducted in 2001. Since then, a number of other investigations have shown that lysins have the potential to be used as antibacterial agents against systemic illnesses (45). In pre-clinical studies, *S. pneumoniae*, the cause of pneumonia, acute otitis media (AOM), septicemia, bronchitis, and meningitis, was successfully eradicated using the pneumococcal lysins Cpl-1 and Pal (46). Ply3626 is a lysin that has activity against the bacterium *C. perfringens*, which can result in food poisoning, necrotic enteritis, and gas gangrene. Human infections with *C. perfringens* may be treated using this enzyme as an antibacterial (46). PlyPH, a putative lysin, has been proven in *in vitro* and *in vivo* experiments to be effective against *B. anthracis*. These lysins have the potential to be used for *B. anthracis* infection diagnosis and treatment (47).

## Conclusion

A novel class of antibiotics called lysins is used to treat bacterial infections. Lysins are recombinant enzymes produced from bacteriophages that directly breakdown the bacterial cell wall, quickly killing the organism. For traditional antibiotics to work, bacterial cell division and metabolism must take place (i.e., cell death or cessation of growth). However, lysins are fundamentally different and quickly eliminate germs upon contact. Peptidoglycan, a crosslinked network of proteins and carbohydrates that makes up the bacterial cell wall, is bound and broken down by lysins. Lysins sever bonds within the peptidoglycan after adhering to it, maintaining the structural stability of the cell wall and the bacterial survival (48).

The current research is the first of its type to determine a therapeutic’s possible characteristics lead, intended for use in developing phage-derived proteins as a potential drug to treat multidrug resistant *A. baumannii*. This *in silico* research is the first step in developing a potent drug that will be anti-fungal, have an anti-biofilm property and possess less toxicity. In the near future when antibiotics will fall short in treating bacterial infections, lysins will show some light in treating such infections (47).

## Data availability statement

Publicly available datasets were analyzed in this study. This data can be found here: Uniprot accession – A0A0P0IE11, A0A7G3T113, L7TMD3, L7TK19, A0A3Q9R756, A0A346FIJ7, A0A6B9STC3, and A0A0B5L1J1.

## Author contributions

AN collected literature data, did *in silico* studies, wrote the manuscript, and tabulated the tables. RY analysed the data and did *in silico* studies. AS wrote the manuscript and critically reviewed and finalized the manuscript. All authors contributed to the article and approved the submitted version.
