# Correction: Phage derived lytic peptides, a secret weapon against *Acinetobacter baumannii*—An *in silico* approach

**DOI:** 10.3389/fmed.2025.1642178

**Published:** 2025-06-27

**Authors:** Abhishek Nandy, Ruchi Yadav, Aditi Singh

**Affiliations:** Amity Institute of Biotechnology, Amity University Uttar Pradesh, Lucknow, India

**Keywords:** endolysin, antimicrobial peptides, biofilm, anti-fungal, *Acinetobacter baumannii*, AMP

The name of the first author needs to be corrected. The author is spelled as “Abhishek Nandi”. The correct name is “Abhishek Nandy.”

The original version of this article has been updated.

